# Effect of T3 Spinal Contusion Injury on Upper Urinary Tract Function

**DOI:** 10.1089/neur.2022.0014

**Published:** 2022-04-26

**Authors:** Jason H. Gumbel, Charles H. Hubscher

**Affiliations:** ^1^ Department of Anatomical Sciences and Neurobiology, University of Louisville, Louisville, Kentucky, USA.; ^2^ Kentucky Spinal Cord Injury Research Center, University of Louisville, Louisville, Kentucky, USA.

**Keywords:** hypothalamus, kidney, natriuretic peptides, polyuria, suprachiasmatic nucleus, vasopressin

## Abstract

Spinal cord injury (SCI) significantly impacts many systems attributable to disrupted autonomic regulation of the body. Of these disruptions, excessive production/passage of urine (polyuria) has been understudied. Pre-clinical animal studies investigating SCI-induced polyuria have been carried out in T8–T10 spinal-level contusive injuries, which directly impacts both supraspinal sympathetic inputs to the spinal circuitry mediating kidney function as well as local networks including pre-ganglionic sympathetic fibers to the kidney. The current study utilizes a higher-level (T3) contusion to narrow the potential source(s) of damage that induce(s) polyuria. Metabolic cage 24-h urine collections demonstrated that, starting 1 week post-SCI and lasting chronically through 6 weeks post-SCI, T3 contused adult male rats had a significant increase in void volume relative to pre-injury and surgical sham controls. Subsequent examination of previously identified biomarkers revealed levels reflecting the presence of polyuria. For example, urine atrial natriuretic peptide levels were significantly increased at 6 weeks post-SCI compared to baseline, and serum arginine vasopressin (AVP) levels were significantly decreased. Further, there was a significant decrease post-injury relative to shams in the number of AVP-labeled cells within the suprachiasmatic nucleus, a hypothalamic region responsible for significant disruptions of circadian rhythmicity post-SCI, including loss of the diurnal variation of AVP production, which clinical studies have identified as contributing to the emergence of nocturia after SCI. Together, the current results demonstrate that SCI-induced polyuria is present after a T3-level SCI, indicating that damage of descending supraspinal circuitries precipitates dysfunction of homeostatic mechanisms involved in salt and water balance.

## Introduction

Spinal cord injury (SCI) is a progressive injury that significantly impacts multiple systems affecting quality of life, including motor, respiratory, cardiovascular, gastrointestinal, and bladder/urinary tract functions.^1–5^ The presence of SCI-induced polyuria increases the number of daily catheterizations, especially at night (disrupting sleep), which raises the risk of developing genitourinary infections, a leading cause for hospitalizations in the SCI population.^6^ SCI-induced polyuria has been shown to be present in both pre-clinical SCI animal models and clinically,^7–10^ regardless of severity^7^ or completeness^11^ of injury.

Under normal physiological conditions, systemic arginine vasopressin (AVP) and blood pressure decreases at nighttime, slowing the production of urine and allowing for uninterrupted sleep. Previous clinical findings have reported a significant lack in diurnal variation (fluctuations between day and night) for urine output and serum AVP levels in the SCI population.^12^ Production of AVP occurs within the supraoptic nucleus (SON), paraventricular nucleus (PVN), and suprachiasmatic nucleus (SCN) of the hypothalamus and functions as an antidiuretic to retain water in response to hyperosmolality or low blood pressure.^13^ The SCN, which is a main regulator of circadian control, including sleep/wake cycles, was recently shown, in a T9-level contusion rat model, to contain significantly fewer AVP-labeled cells beginning as early as 14 days post-injury (dpi).^14^

However, significant up- or downregulation at various time points, beginning at 7 days post-injury, have been shown for kidney natriuretic peptide receptor-A (NPRA), atrial natriuretic peptide (ANP), kidney vasopressin-2 receptor (V2R), kidney aquaporin-2 (AQP2) channels, and kidney epithelial sodium channels (ENaC; β and γ but not α subunits), suggesting that both central and peripheral mechanisms are involved in the development and maintenance of polyuria.^9,14,15^ Together, these biomarkers are key for both water/sodium and cardiovascular homeostasis. Specifically, ANP causes an increase of sodium/water excretion opposite to that of AVP. Note that ANP inhibits the antidiuresis effect of AVP within the kidney. Additional background information on these SCI-induced, polyuria-associated biomarkers is reviewed elsewhere.^13^

Autonomic dysregulation occurs after SCI because of disruption of supraspinal sympathetic pathways descending from various brain regions and/or local spinal networks and/or pre-ganglionic sympathetic outputs. The kidneys, which regulate and balance the body's water and metabolite content (filtration from blood as urine), receives sensory innervation from T9 to L2 ipsilateral dorsal root ganglia and sympathetic supply from post-ganglionic neurons located within the celiac ganglion,^16^ which has been traced to pre-ganglionic neurons at the T4–T13 spinal levels in rats.^17^ Note that there is limited evidence for specific parasympathetic supply to the kidneys.^18,19^ Sympathetic pre-ganglionic fibers receive input from both intra- and supraspinal neurons.^20^ The descending supraspinal inputs to sympathetic pre-ganglionic neurons arise from the rostral ventrolateral medulla, rostral ventromedial medulla, caudal raphe nuclei, A5 region, and paraventricular nucleus of the hypothalamus.^21,22^

Subsequent to disruption of supraspinal drive post-SCI, spinal autonomic interneurons undergo plasticity and are key regulators of spinal sympathetic preganglionic circuitry.^23^ Note also that the vagal supply of the viscera has been shown to undergo neurochemical plasticity post-SCI, a finding with implications for visceral homeostatic mechanisms and nociceptive signaling after chronic injury.^24^

Our pre-clinical research on SCI-induced polyuria to date has focused on a T9-level injury,^7–9^ which disrupts both supraspinal and local pre-ganglionic/interneuronal sympathetic spinal circuitries to the kidney and surrounding vasculature. The goal of the current investigation was to determine whether damage of supraspinal sympathetic control to the kidney and/or disruption of local spinal circuitry surrounding a T9-level SCI mediate the development and maintenance of polyuria by examining outcomes after a T3-level spinal contusion, which is just above the sensory/interneuron/pre-ganglionic supply to/from the kidneys. Of potential relevance are findings showing that cervical (C2–C8)-level injuries in humans generate higher urinary outputs than those having T1- to L1-level injuries, specifically at night,^10^ in addition to being at greater risk for autonomic dysreflexia.^25^

## Methods

### Animals

All animal experimental procedures and protocols were reviewed and approved by the Institutional Animal Use and Care Committee (IACUC) at the University of Louisville School of Medicine (Louisville, KY) and carried out according to National Institutes of Health (NIH) guidelines. For this study, 18 adult male Wistar rats (∼250 g) were individually housed with a 12:12-h light-dark cycle. Animals either received a T3 SCI (*n* = 12) or sham surgery (laminectomy with no contusion injury, *n* = 6). Note that only male rats were used for this study because they represent clinically the vast majority of the SCI population.

### Spinal cord injury

After pre-injury baseline assessments (see below), animals were anesthetized with an intraperitoneal injection of ketamine (80 mg/kg; Ketoset^®^; Fort Dodge Laboratories, Fort Dodge, IA) and xylazine (10 mg/kg; AnaSed; Lloyd Laboratories, Shenandoah, IA). To assure a deep anesthetic plane, both toe pinch and orbital reflexes were monitored. The surgical area was shaved and cleansed with 4% chlorhexidine scrub (Henry Schein, Melville, NY), and sterile ocular lubricant (OptixCare; Aventix, Burlington, Ontario, Canada) was applied. A T2 laminectomy was performed to expose the T3-level of spinal cord. Contusions were performed at a force that yields a moderate-severe contusion,^7,25^ using an Infinite Horizon (IH) impactor (Precision Systems and Instrumentation LLC, Fairfax Station, VA) per established T9 protocols (215-kdyne force with no dwell time).^7,26^

The muscular layer and skin were closed with a 4-0 surgical suture (Ethicon; Ethicon Inc. Somerville, NJ) and surgical wound clips. Antibiotic (penicillin G; PenJect; Henry Schein Animal Health) and analgesic (meloxicam; Eloxiject; Henry Schein Animal Health) were injected subcutaneously per established post-operative care procedures (0.1 and 0.2 mL per animal, respectively).^9,27,28^ Physiological saline was also administered just before surgery (5 mL) and immediately after contusion (5 mL) to account for fluid loss during surgery (note that all rat groups received these one-time supplemental fluids, including the surgical sham group). This procedure from our lab is available in video journal format online.^28^

Initially post-contusion, rats presented with flaccid bladder paralysis resulting in an areflexic bladder, for which manual bladder emptying was performed with the Crede maneuver three times daily until individual animals reached reflexive bladder function (by 6 dpi^29,30^). Urine was collected in a plastic tube, and volumes were measured as an additional indicator for consistency of the IH impactor, but only on day 4, per one of our previous studies with T9 level of injury showing peak dysfunction during the initial areflexic period at 3–4 days post-SCI.^7,30^ Note that once animals reached reflexive bladder emptying, manual bladder expression was no longer used.

### Metabolic cage data collection

For 24-h metabolic cage data collection, animals were placed in a six-station Comprehensive Lab Animal Monitoring System (CLAMS; Columbus Instruments, Columbus, OH) to monitor 24-h urine output volume and drink volume according to established protocols.^7,9,31^ To acclimate animals to the CLAMS unit, pre-injury baseline data were collected twice in 1 week, but only the second 24-h period was used for analysis. Metabolic cage assessments were then carried out once-weekly.^9,15,28^ Data from the CLAMS unit were exported to an excel spreadsheet where data abstraction was analyzed blinded to time point. Calculation of 24-h urine volumes was obtained by taking the sum of each void event ≥0.2 g recorded by the sensor within the 24-h time frame. Total drink volume was recorded through the CLAMS volumetric drink monitor.

### Blood and urine sample collection

The lateral tail vein was used for blood/serum sample collection at pre-injury and end of the study (6 weeks post-injury [wpi]) time points. Isoflurane was used to anesthetize animals. The base of the tail was shaved for better visualization of lateral tail veins. Animals were then placed on a heating pad where an 18-g needle was used to puncture either of the lateral tail veins, and 0.5–0.7 mL of blood was collected into serum separator tubes (BD microcontainer; Becton, Dickinson and Co., Franklin Lakes, NJ). Bleeding was stopped by applying light pressure with 2 × 2 in gauze or, when necessary, styptic powder with benzocaine (Kwik-Stop; ARC Laboratories). Blood samples were then centrifuged at 14,000 rpm for 15 min, and serum was collected and stored at −20°C for future analysis. Urine samples were collected from 24-h metabolic cages. Urine samples were then centrifuged at 14,000 rpm for 15 min. Urine was aliquoted into 2-mL tubes and stored at −20°C until used for analysis.

### Locomotor assessment

For locomotor assessment, the Basso-Beattie-Bresnahan (BBB) open-field locomotor test^32^ was performed weekly on each rat as well as the day preceding terminal time point. A single score per animal was obtained by averaging the left and right hindlimb BBB score assigned by two experimenters blinded to time-point status (post-SCI). These scores are used as an additional indicator of lesion severity and spontaneous recovery, per our previously published data, where, taken together with white matter sparing (WMS) and 4-day residual volume, injury outliers can be identified.^7^

### Tissue collection and histology

Animals underwent transcardial perfusions with exsanguination solution (heparinized phosphate-buffered solution: 1 mg of heparin per 1 L of phosphate-buffered saline), followed by 4% paraformaldehyde at the end of the study (6 wpi). Multiple tissues were removed, but only the brain (for hypothalamus) and spinal cord (lesion site) were used for this study. The brain and spinal cord were then submerged in 4% paraformaldehyde for storage at 4°C for at least 24 h, then moved to a 30% sucrose solution and stored at 4°C until being sectioned using a cryostat (Leica CM 1850; Leica Microsystems, Nussloch, Germany).

For hypothalamus tissue, serial coronal sections of 25 μm were cut, assuring that each glass slide contained three sections ≥75 μm apart to avoid double counting of cells to be stained for analysis. Tissue was stained with anti-AVP primary antibody (ab39363; Abcam, Cambridge, MA) to visualize and quantify the number of AVP-labeled cells in the SCN, a region involved in the diurnal variation of AVP.^33^ Slides were washed in 1 × phosphate-buffered saline before and after antigen retrieval (Enzo Life Sciences, Farmingdale, NY), then incubated in 0.3% H_2_O_2_ for peroxide blocking. Blocking was performed using SuperBlock (ThermoFisherScientific, Waltham, MA), and primary antibody was diluted at 1:1000 in 4% normal goat serum, applied to slides, and incubated at 4°C overnight. Secondary antibody (fluorescent-conjugated goat antirabbit; Alexa Fluor 488; ThermoFisherScientific) was diluted in 4% normal goat serum and incubated for 1 h at room temperature. Slides were washed, incubated with 4′,6-diamidino-2-phenylindole (ThermoFisherScientific), then cover-slipped before imaging and analysis. The number of AVP-positive cells was quantified using ImageJ (NIH). First, the area of the SCN was outlined, and AVP-labeled cells with an intensity threshold at least 1.5 times above background level were counted to obtain the cells per area for quantification. At least four different sections of nuclei per animal were averaged together for analyses, per our published protocols.^14^

Histology of the spinal lesion site for WMS was carried out as previously described.^9,31,34^ The spinal cord lesion site, including approximately two levels above and below, was sectioned in the coronal plane at a 20-μm thickness and stained with Luxol fast blue and cresyl violet. The lesion epicenter and WMS was captured and analyzed using Spot Advanced software (Diagnostic Instruments, Sterline Heights, MI) and a Nikon E400 microscope (Nikon Corporation, Tokyo, Japan). Percentage of WMS was calculated by dividing intact white matter at the lesion epicenter by intact white matter rostral and caudal to the injury site. An average of two areas ≥2 mm both rostral and caudal was used for intact white matter.

### *Enzyme-linked immunosorbent* assay

Urinary ANP was measured using an Enzyme Immunoassay Kit (catalog no.: K026-H1; Arbor Assays, Ann Arbor, MI), and creatinine was measured using the DetectX Urinary Creatinine Detection Kit (catalog no.: K002-H5; Arbor Assays) for pre-injury and 6-wpi time points.^9^ Urine samples were diluted at 1:5 for ANP and 1:20 for creatinine and then plated in a 96-well plate in duplicate. ANP and creatinine plates were read at a 450-nm optical density using SoftMax Pro software (Molecular Devices, LLC. San Jose, CA). Urinary creatinine levels were used to control for differing urine concentrations per ANP enzyme-linked immunosorbent assay (ELISA) kit instructions. To obtain accurate urinary ANP levels, ANP levels were divided by creatinine levels.

Baseline and terminal levels of serum AVP were determined using an arginine vasopressin ELISA kit (catalog no.: OKEH02585; Aviva Systems Biology, San Diego, CA). Stored serum samples (see above) were diluted at 1:5, and ELISA was carried out according to kit instructions.

### Statistical analysis

Two-way analysis of variance analyses were performed to compare T3 to sham animals for metabolic cage, ELISA, and AVP-labeled cell-count data using SigmaStat software (v3.5; Systat Software, Palo Alto, CA), where significance was determined for *p* < 0.05.

For ELISA analysis, data files for AVP, ANP, and creatinine were exported from SoftMax Pro to Microsoft Excel (Microsoft Corporation, Redmond, WA). Averages of samples and standards were used to create a standard curve for determination of protein concentration. Because of combined variability and sample size, terminal protein concentrations were compared to their normalized baseline level for statistical analysis. Signed-ranks tests were used to compare normalized baseline to terminal protein concentrations, where *p* < 0.05 was considered statistical significance.

## Results

Data were obtained from a total of 17 adult male Wistar rats (T3 SCI, *n* = 11; surgical sham, *n* = 6; 1 animal died because of complications of injury). Before contusion injuries, pre-injury baseline data were collected, which included 24-h metabolic cages, blood draws, and BBB assessments. To confirm the consistency of contusions across all animals, the mean force and displacement generated by the IH impactor were evaluated. In addition, day 4 residual volume, BBB scores, and WMS were evaluated. Mean data are summarized in Table 1. Histological reconstruction of the lesion epicenters revealed a mean percent WMS for the T3 SCI group as 21.2% ± 1.7%. *Post-hoc* analyses for outliers (Grubbs’ test) were done and none were found in the SCI (or sham) animal group, indicating consistency of the contusion injury.

**Table 1.** Injury Parameters and Assessment Outcome Values

Group	*n*	Injury force (kdyne)	Displacement (μm)	4 dpi residual urine volume (mL)	7 dpi BBB	6 wpi BBB
T3 SCI	11	218.8 ± 7.5	1310 ± 175	0.35 ± 0.21	8.80 ± 1.60	11.0 ± 0.0
Sham	6	—	—	—	20.0 ± 0.0	20.30 ± 0.82

Values indicated are means ± standard deviation; The T3 + SCI values for both 7 dpi BBB and 6 wpi BBB were significantly different from sham values (*p* < 0.05).

SCI, spinal cord injury; dpi, days post-injury; wpi, weeks post-injury; BBB, Basso-Beatie-Bresnahan locomotor scale.

### 24-h urine and drink volumes

**FIG. 1.**
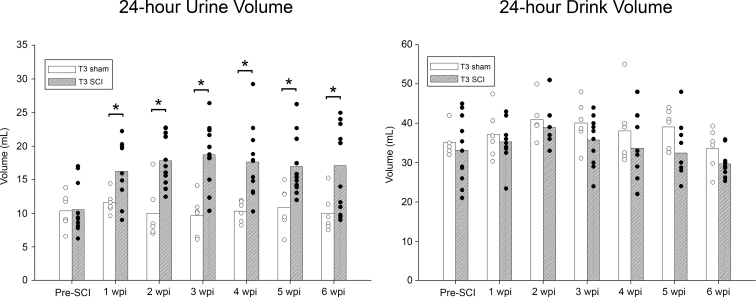
Metabolic cage data summary. Total 24-h urine volume output (left graph) demonstrates a statistically significant increase in urine production per passage at 1 wpi, lasting through 6 wpi, compared to pre-injury baseline volume and sham volumes. Total 24-h drink volume (right graph) indicates an absence of statistically significant changes in water intake across all time points relative to pre-injury and sham groups (*p < 0.05; T3 SCI, *n* = 11; sham, *n* = 6). SCI, spinal cord injury; wpi, weeks post-injury.

To determine the presence of SCI-induced polyuria, metabolic cages were used to quantify 24-h urine volumes. The data are presented in Figure 1. Compared to both sham and pre-injury baseline volumes, T3 animals revealed a statistically significant increase in 24-h urine volumes, starting at 1 wpi, and was present at every other time point. Drink volumes were not significantly different between T3 SCI animals and shams at any time point, nor were any time points statistically significantly different from each other. Although average void volume increased whereas drink volume did not significantly change, animals did not present with any visible signs of dehydration (e.g., skin turgidity). Further, body weight did not decrease over time, but instead increased, which is likely attributable to limited mobility post-SCI.

### Atrial natriuretic peptide and arginine vasopressin

Both urinary ANP and serum AVP were investigated using ELISA, because they are key regulators of cardiovascular and water/solute homeostasis. Serum levels of AVP were significantly lower in T3 SCI animals compared to normalized pre-injury baseline levels at 6 wpi (0.62 ± 0.08 fold-change). Additionally, urinary ANP was significantly elevated (average 1.62 ± 0.07 fold-change) at 6 wpi compared to pre-injury baseline in T3 SCI animals.

### Arginine vasopressin labeling in hypothalamus

**FIG. 2.**
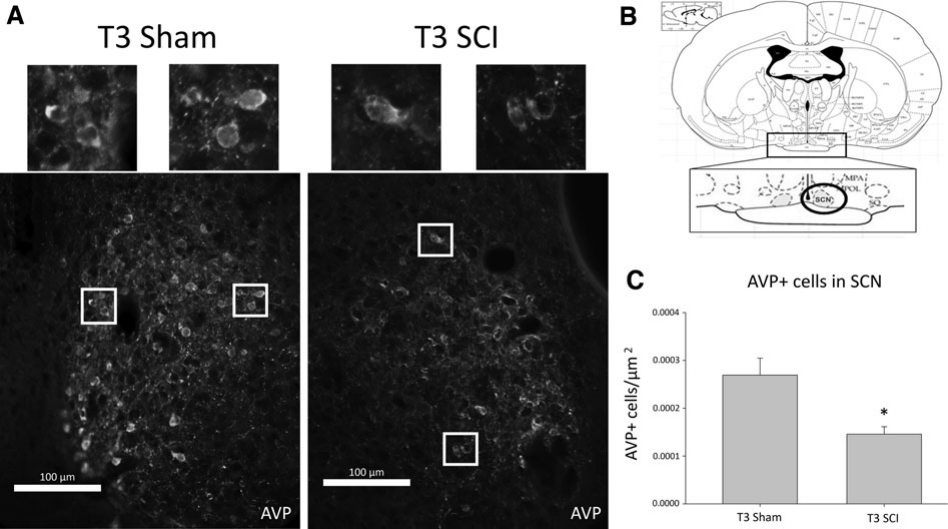
Suprachiasmatic nucleus (SCN) immunohistochemistry. A representative section showing AVP-labeled cells at 400 × in the SCN in both T3 sham and T3 SCI animals (**A**). In (**B**), a modified plate from the Rat Brain Atlas illustrates the location of the SCN within the hypothalamus.^48^ Average quantified AVP-labeled cells/μm in the SCN was statistically significantly lower in T3 SCI animals compared to sham controls (**C;** *p* < 0.05; T3 SCI, *n* = 5; T3 sham, *n* = 4). Values (shown in C) represent means; error bars represent standard error of means. AVP, arginine vasopressin; SCI, spinal cord injury.

The number of AVP-producing cells in the SCN was quantified and revealed that the average AVP-positive cells/μm was significantly lower in T3 SCI animals than T3 shams (Fig. 2). Note the integrity of the hypothalamus tissue was not ideal for quantification in several animals, yielding *n* = 5 for T3 SCI and *n* = 4 for T3 sham groups. It is important to note that tissue collection from all animals occurred within the same 2-h time frame, given that AVP production in the SCN is time dependent.^35^

The mean percent WMS for the T3 SCI group was 21.2% ± 1.7%. As a further analysis, a Pearson correlation was conducted on WMS and terminal (6 wpi) 24-h urine volumes. There was no significant correlation between WMS and 24-h urine volume (correlation coefficient = 0.34; *p* > 0.05).

## Discussion

Results of the current T3-level contusion SCI study demonstrate a significant increase in 24-h urine volumes at 1 through 6 wpi. Given that the spinal sympathetic supply to the kidney is mostly intact below a T3 level of injury, the development and maintenance of SCI-induced polyuria was likely attributable to damage of descending supraspinal circuitries, which likely disrupts fluid and metabolite homeostasis, precipitating plasticity within the kidney itself, such as previously observed with fluctuations in the relative expression of receptors and channels^9,14,15^ as well as central regions, including those containing AVP-producing neurons within the hypothalamus.^14^ Notably, daily urine volumes in T3 SCI animals were equivalent to T9 SCI animals of past studies.^9,14^ Further, similar to previous findings with a T9 SCI, there were no significant changes in drink volumes at any of the time points.^7,9^ Stability in drink volume from pre-SCI through chronic SCI, taken together with an increase in urine production per passage, is indicative of the extent to which systemic body water/solute balance is severely disrupted after SCI. Further studies investigating sources of this disbalance are in progress.

It is important to note that 4-dpi residual volume was carried out as one of our standard data points, in the same fashion WMS and BBB assessments are, which is a further indicator of consistency of the contusion injury. In a T9 injury, 4-dpi residual volumes are much greater than the volumes reported here. This finding is likely because a T9 injury is in closer proximity to the thoracolumbar cord containing the sympathetic circuitry that mediates lower urinary tract function. Thus, a T3-level injury would likely recover from the flaccid bladder paralysis that occurs in the acute stage post-contusion in a shorter time frame. Note that this T3/T9 difference is not likely attributable to overall severity, given that both BBB and WMS in these T3 rats are consistent with a moderate-severe T9-level injury.

Also consistent with past findings in T9 contused rats was a decrease in serum AVP and an increase in urinary ANP after chronic T3 SCI.^8,9^ Both ANP and AVP are crucial for body water/solute homeostasis, and the change of their alteration observed in this and recent studies are in the direction that would result in polyuria.^9,36^ The similar findings with T3 SCI animals in relation to T9 SCI animals previously reported^9,14^ suggest that these mechanisms are present regardless of level of injury.

An additional focus of this study was the SCN of the hypothalamus, because previous data from our lab suggest that there are no consistent changes in numbers of AVP-labeled cells within the SON or PVN after chronic T9 SCI and, clinically, diurnal variations of AVP are lost. As noted with T9 lesions, there were significantly fewer AVP-labeled cells in the SCN of the hypothalamus in T3 SCI rats.^14^ The SCN is one of three nuclei in the hypothalamus where AVP is made^37^ and is heavily involved in circadian control of both humans and rodents.^38,39^ Recent studies have reported that circadian rhythmicity is significantly disrupted by SCI.^12,40,41^

However, we have previously shown, with a T9-level injury, that significant polyuria is present in both the quiescent and active phase of the light-dark cycle in rats,^7^ suggesting that persons with SCI may also experience polyuria at both day/night cycles as well. Thus, a prospective study with appropriate controls (age, weight, blood pressure, etc.) measuring both drink and urine volumes on an hourly basis may be beneficial to elucidate this effect. Clinically, investigations have found reduced sodium conservation in SCI persons with higher levels of injury^42^ and a greater imbalance in day/night urine flow rates among those with cervical region versus all other levels of SCI.^10^ Further investigations are clearly needed to better understand the role of circadian rhythms in fluid homeostasis.

The mechanisms behind SCI-induced polyuria are not yet fully understood. However, recent studies have shown that AVP, ANP and their associated receptors, V2R and NPRA, plus water channel AQP2 and ENaC, are significantly altered after SCI in rats.^8,9^ The significant decrease in AVP, specifically at night, has also been shown in the clinical setting.^12,43^ Together, the changes in these hormone peptides/receptors are in the direction that would indicate an increase in urine production and require further investigation for the elucidation of SCI-induced polyuria mechanisms.

The findings reported here suggest that polyuria is induced from damage of supraspinal inputs to pre-ganglionic sympathetic neurons supplying the kidneys, although secondary systemic responses related to inflammatory processes and/or perturbation of immune functions are likely contributing factors. These disturbances, as further indicated by changes in a variety of biomarker of fluid homeostasis, including ANP, may also be further complicated by alterations in cardiovascular dynamics with higher levels of SCI. The spacing between T3 and T9 is not likely wide enough to detect significant differences in 24-h urine volumes, or ANP/AVP concentrations, as is evident clinically in the population with cervical-level injuries.^42^

Blood pressure and urine production work in conjunction to keep each other balanced. There are several mechanisms that work to maintain water/solute homeostasis by affecting blood pressure. For example, studies focusing on hypertensive models, both animal and clinical, have demonstrated that denervation of the sympathetic supply to the kidneys results in abolishment of hypertension.^44,45^ Thus, alterations of neural control of the kidney is likely a contributing factor to orthostatic hypotension, a common occurrence post-SCI.^46^ Multiple studies have suggested that a higher level of injury may result in further impairment of cardiovascular and upper urinary dysfunction.^42,47^ Therefore, a future study investigating an even higher level of SCI (cervical) would likely be beneficial in elucidating the extent that level of injury has upon SCI-induced polyuria.

### Ethical approval

All animal experimental procedures and protocols were reviewed and approved by the IACUC at the University of Louisville School of Medicine and carried out according to NIH guidelines.

## Abbreviations Used

ANP: atrial natriuretic peptide
AQP2: aquaporin-2
AVP: arginine vasopressin
BBB: Basso-Beatie-Bresnahan
CLAMS: Comprehensive Lab Animal Monitoring System
dpi: days post-injury
ELISA: enzyme-linked immunosorbent assay
ENaC: epithelial sodium channel
IACUC: Institutional Animal Use and Care Committee
IH: Infinite Horizon impactor
NIH: National Institutes of Health
NPRA: natriuretic peptide receptor-A
PVN: paraventricular nucleus
SCI: spinal cord injury
SCN: suprachiasmatic nucleus
SON: supraoptic nucleus
V2R: vasopressin-2 receptor
WMS: white matter sparing
wpi: weeks post-injury

## References

[1] Anderson K, Borisoff J, Johnson R, et al. The impact of spinal cord injury on sexual function: concerns of the general population. Spinal Cord 2007;45(5):328–337. 10.1038/sj.sc.3101977 17033620

[2] Cruz CD, Cruz F. Spinal cord injury and bladder dysfunction: new ideas about an old problem. TheScientificWorldJournal 2011;11:214–234. 10.1100/tsw.2011.26 PMC5720001 21258763

[3] Steadman CJ, Hubscher CH. Sexual function after spinal cord injury: innervation, assessment, and treatment. Curr Sex Health Rep 2016;8(2):106–115.

[4] Krassioukov A, Claydon VE. The clinical problems in cardiovascular control following spinal cord injury: an overview. Prog Brain Res 2006;152:223–229. 10.1016/S0079-6123(05)52014-4 16198703

[5] Anderson KD. Targeting recovery: priorities of the spinal cord-injured population. J Neurotrauma 2004:21(10);1371–1383. 10.1089/neu.2004.21.1371 15672628

[6] Cardenas DD, Hoffman JM, Kirshblum S, et al. Etiology and incidence of rehospitalization after traumatic spinal cord injury: a multicenter analysis. Arch Phys Med Rehabil 2004;85(11):1757–1763. 10.1016/j.apmr.2004.03.016 15520970

[7] Ward PJ, Hubscher CH. Persistent polyuria in a rat spinal contusion model. J Neurotrauma 2012;29(15):2490–2498. 10.1089/neu.2012.2402 PMC3471123 22708983

[8] Montgomery LR, Hubscher CH. Altered vasopressin and natriuretic peptide levels in a rat model of spinal cord injury: implications for the development of polyuria. Am J Physiol Renal Physiol 2018;314:F58–F66. 10.1152/ajprenal.00229.2017 28877880

[9] Gumbel JH, Montgomery LR, Yang CB, et al. Activity-based training reverses spinal cord injury-induced changes in kidney receptor densities and membrane proteins. J Neurotrauma 2020;37(3):555–563. 10.1089/neu.2019.6670 31456470

[10] Goh MY, Millard MS, Wong EC, et al. Comparison of diurnal blood pressure and urine production between people with and without chronic spinal cord injury. Spinal Cord 2018;56(9):847–855. 10.1038/s41393-018-0081-3 29500404

[11] Viaene A, Denys MA, Goessaert AS, et al. Evaluation of the occurrence and diagnose definitions for nocturnal polyuria in spinal cord injured patients during rehabilitation. Eur J Phys Rehabil Med 2017;55(1):40–46. 10.23736/S1973-9087.17.04851-1 29099160

[12] Kilinç S, Akman MN, Levendoglu F, et al. Diurnal variation of antidiuretic hormone and urinary output in spinal cord injury. Spinal Cord 1999;37(5):332–335. 10.1038/sj.sc.3100814 10369169

[13] Gumbel JH, Hubscher CH. Hormonal Events and Spinal Cord Injury: A Focus on Vasopressin and Natriuretic Peptide. In: Cellular, Molecular, Physiological and Behavioral Aspects of Spinal Cord Injury. (Rajendram R, Preedy V, Martin C, eds.) Elsevier Academic; 2022. [In press.]

[14] Gumbel JH, Yang CB, Hubscher CH. Timeline of changes in biomarkers associated with spinal cord injury-induced polyuria. Neurotrauma Rep 2021;2(1):462–475. 10.1089/neur.2021.0046 PMC8655813 34901942

[15] Montgomery LR, Hubscher CH. Altered vasopressin and natriuretic peptide levels in a rat model of spinal cord injury: implications for the development of polyuria. American Journal of Physiology-Renal Physiology. 2017;314(1):F58–F66. 10.1152/ajprenal.00229.2017 28877880

[16] Gattone V II, Marfurt CF, Dallie S. Extrinsic innervation of the rat kidney: a retrograde tracing study. Am J Physiol Renal Physiol 1986;250(2):F189–F196. 10.1152/ajprenal.1986.250.2.F189 3753828

[17] Strack A, Sawyer W, Marubio L, Loewy A. Spinal origin of sympathetic preganglionic neurons in the rat. Brain Res 1988;455(1):187–191. 10.1016/0006-8993(88)90132-1 3416186

[18] Maeda S, Kuwahara-Otani S, Tanaka K, et al. Origin of efferent fibers of the renal plexus in the rat autonomic nervous system. J Vet Med Sci 2014;76(5):763–765. 10.1292/jvms.13-0617 PMC4073349 24430660

[19] Kirkpatrick JJ, Foutz S, Leslie SW. Anatomy, abdomen and pelvis, kidney nerves. StatPearls [Internet]. StatPearls Publishing LLC: Treasure Island, FL: 2020. 29083631

[20] Llewellyn-Smith IJ, Weaver LC, Keast JR. Effects of spinal cord injury on synaptic inputs to sympathetic preganglionic neurons. Prog Brain Res 2006;152:11–26. 10.1016/S0079-6123(05)52001-6 16198690

[21] Strack A, Sawyer W, Hughes J, et al. A general pattern of CNS innervation of the sympathetic outflow demonstrated by transneuronal pseudorabies viral infections. Brain Res 1989;491(1):156–162. 10.1016/0006-8993(89)90098-x 2569907

[22] Sved AF, Cano G, Card JP. Neuroanatomical specificity of the circuits controlling sympathetic outflow to different targets. Clin Exp Pharmacol Physiol 2001;28(1-2):115–119. 10.1046/j.1440-1681.2001.03403.x 11153526

[23] Schramm LP. Spinal sympathetic interneurons: their identification and roles after spinal cord injury. Prog Brain Res 2006;152:27–37. 10.1016/S0079-6123(05)52002-8 16198691

[24] Herrity AN, Petruska JC, Stirling DP, et al. The effect of spinal cord injury on the neurochemical properties of vagal sensory neurons. Am J Physiol Regul Integr Comp Physiol 2015;308(12):R1021–R1033. 10.1152/ajpregu.00445.2014 PMC4469926 25855310

[25] Curt A, Nitsche B, Rodic B, et al. Assessment of autonomic dysreflexia in patients with spinal cord injury. J Neurol Neurosurg Psychiatry 1997;62(5):473–477. 10.1136/jnnp.62.5.473 PMC486854 9153603

[26] Scheff SW, Rabchevsky AG, Fugaccia I, et al. Experimental modeling of spinal cord injury: characterization of a force-defined injury device. J Neurotrauma 2003;20(2):179–193. 10.1089/08977150360547099 12675971

[27] Steadman CJ, Vangoor SS, Hubscher CH. Kinematic analysis of penile reflexes in a rat model of spinal cord injury. Asian J Androl 2021;23(1):30–35. 10.4103/aja.aja_1_20 PMC7831836 32341209

[28] Gumbel JH, Steadman CJ, Hoey RF, et al. Activity-based training on a treadmill with spinal cord injured Wistar rats. J Vis Exp 2019;(143):e58983. doi: 10.3791/58983. 30735203

[29] Ferrero SL, Brady TD, Dugan VP, et al. Effects of lateral funiculus sparing, spinal lesion level, and gender on recovery of bladder voiding reflexes and hematuria in rats. J Neurotrauma 2015;32(3):200–208. 10.1089/neu.2013.3247 PMC4298755 25137571

[30] Holmes GM, Hubscher CH, Krassioukov A, et al. Recommendations for evaluation of bladder and bowel function in pre-clinical spinal cord injury research. J Spinal Cord Med 2020;43(2):165–176. 10.1080/10790268.2019.1661697 PMC7054945 31556844

[31] Ward PJ, Herrity AN, Smith RR, et al. Novel multi-system functional gains via task specific training in spinal cord injured male rats. J Neurotrauma 2014;31(9):819–833. 10.1089/neu.2013.3082 PMC3996943 24294909

[32] Basso DM, Beattie MS, Bresnahan JC. A sensitive and reliable locomotor rating scale for open field testing in rats. J Neurotrauma 1995;12(1):1–21. 10.1089/neu.1995.12.1 7783230

[33] Mieda M, Ono D, Hasegawa E, et al. Cellular clocks in AVP neurons of the SCN are critical for interneuronal coupling regulating circadian behavior rhythm. Neuron 2015;85(5):1103–1116. 10.1016/j.neuron.2015.02.005 25741730

[34] Steadman CJ, Hoey RF, Montgomery LR, et al. Activity-based training alters penile reflex responses in a rat model of spinal cord injury. J Sex Med 2019;16(8):1143–1154. 10.1016/j.jsxm.2019.05.018 31277969

[35] Kalsbeek A, Buijs R, Engelmann M, et al. In vivo measurement of a diurnal variation in vasopressin release in the rat suprachiasmatic nucleus. Brain Res 1995;682(1-2):75–82. 10.1016/0006-8993(95)00324-j 7552330

[36] Zerbe RL, Robertson GL. A comparison of plasma vasopressin measurements with a standard indirect test in the differential diagnosis of polyuria. N Engl J Med 1981;305(26):1539–1546. 10.1056/NEJM198112243052601 7311993

[37] Sofroniew MV, Weindl A. Projections from the parvocellular vasopressin-and neurophysin-containing neurons of the suprachiasmatic nucleus. Am J Anat 1978;153(3):391–429. 10.1002/aja.1001530305 360814

[38] Meijer J, Rietveld W. Neurophysiology of the suprachiasmatic circadian pacemaker in rodents. Physiol Rev 1989;69(3):671–707. 10.1152/physrev.1989.69.3.671 2664825

[39] Hofman M, Purba J, Swaab D. Annual variations in the vasopressin neuron population of the human suprachiasmatic nucleus. Neuroscience 1993;53(4):1103–1112. 10.1016/0306-4522(93)90493-y 8506022

[40] Gaudet AD, Fonken LK, Ayala MT, et al. Spinal cord injury in rats disrupts the circadian system. eNeuro 2018;5(6):ENEURO.0328-18.2018. doi: 10.1523/ENEURO.0328-18.2018. PMC6325559 30627655

[41] Baschieri F, Guaraldi P, Provini F, et al. Circadian and state-dependent core body temperature in people with spinal cord injury. Spinal Cord 2021;59(5):538–546. 10.1038/s41393-020-0521-8 32681119

[42] Frisbie J. Salt wasting, hypotension, polydipsia, and hyponatremia and the level of spinal cord injury. Spinal Cord 2007;45(8):563–568. 10.1038/sj.sc.3101984 17033618

[43] Szollar SM, Dunn KL, Brandt S, et al. Nocturnal polyuria and antidiuretic hormone levels in spinal cord injury. Arch Phys Med Rehabil 1997;78(5):455–458. 10.1016/s0003-9993(97)90155-6 9161360

[44] Bello-Reuss E, Colindres R, Pastoriza-Munoz E, et al. Effects of acute unilateral renal denervation in the rat. J Clin Invest 1975;56(1):208–217. 10.1172/JCI108069 PMC436571 1141432

[45] Kannan A, Medina RI, Nagajothi N, et al. Renal sympathetic nervous system and the effects of denervation on renal arteries. World J Cardiol 2014;6(8):814–823. 10.4330/wjc.v6.i8.814 PMC4163710 25228960

[46] Claydon VE, Steeves JD, Krassioukov A. Orthostatic hypotension following spinal cord injury: understanding clinical pathophysiology. Spinal Cord 2006;44(6):341–351. 10.1038/sj.sc.3101855 16304564

[47] Teasell RW, Arnold JMO, Krassioukov A, et al. Cardiovascular consequences of loss of supraspinal control of the sympathetic nervous system after spinal cord injury. Arch Phys Med Rehabil 2000;81(4):506–516. 10.1053/mr.2000.3848 10768544

[48] Paxinos G, Watson C. The Rat Brain in Stereotaxic Coordinates. Elsevier Academic: Burlington, MA; 2006.

